# Preparation of oat galactolipid and anti-liver cancer effects of oat galactolipid–modified curcumin-loaded liver targeting vesicle

**DOI:** 10.3389/fphar.2024.1511666

**Published:** 2025-01-08

**Authors:** Huiying Ren, Nuo Chen, Yanqing Liu, Meimei Wu, Jingsong Yan, Mingxiang Chang, Hanmin Li

**Affiliations:** ^1^ Hubei Key Laboratory of Resources and Chemistry of Chinese Medicine, School of Pharmacy, Hubei University of Chinese Medicine, Wuhan, China; ^2^ Hubei Shizhen Laboratory, Wuhan, China; ^3^ Hubei Key Laboratory of Theory and Application Research of Liver and Kidney, Hubei Provincial Hospital of Traditional Chinese Medicine, Wuhan, China; ^4^ Affiliated Hospital of Hubei University of Chinese Medicine, Wuhan, China

**Keywords:** oat galactolipid, curcumin, oat-galactolipid-modified curcumin-loaded liver-targeting vesicle, liver targeting, anti-liver cancer efficacy

## Abstract

**Introduction:**

The mortality rate for liver cancer is extremely high but clinical treatments have not made much progress, so it is necessary to develop anticancer agents with lower toxicities and more effective liver-targeting drug delivery systems (LTDDSs). At present, LTDDSs mediated by the asialoglycoprotein receptor (ASGPR) show excellent effects at improving the liver-targeting and antitumor effects of drugs. However, the galactosyl ligands are typically prepared by chemical synthesis and have some shortcomings. The present work endeavors to explore the influences of plant galactolipids as natural galactosyl ligands for LTDDSs.

**Methods:**

Plant galactolipids were extracted from oat bran, and their characteristics were tested. Then, oat-galactolipid-modified curcumin-loaded liver-targeting vesicles (GCLTVs) and curcumin-loaded vesicles were prepared, which were used in a comparative study of the liver-targeting and liver anticancer effects *in vitro* and *in vivo*.

**Result:**

The experimental results show that the oat galactolipids and GCLTVs were prepared successfully. The hydrophilic–lipophilic balance, acid, ester, and saponification values of the oat galactolipids were 14.89, 47.22, 237.09, and 284.30, respectively. The morphology of the GCLTV was spherical, with an average particle size of 64.47 nm and average potential of −19.73 mV. The optimal proportion of galactolipids in the GCLTVs was selected as 30%. Compared with the curcumin-loaded vesicles, GCLTV uptakes were significantly higher at 1, 2, and 4 h; further, the galactolipid modification significantly improved the liver-targeting capability of the GCLTVs *in vivo*. The inhibitory effects of the GCLTVs on the proliferation of HepG2 cells were significantly higher than those of the curcumin-loaded vesicles after 24 and 48 h. The antitumor effects of GCLTVs *in vivo* based on H&E staining results on liver tissues were stronger than those of the curcumin-loaded vesicles, and the expressions of P53, Bcl-2, and Bax were correspondingly more significant.

**Conclusion:**

The GCLTVs show excellent liver-targeting capabilities *in vitro* and *in vivo*. Compared to the curcumin-loaded vesicles, the cytotoxicity and anticancer effects of the GCLTVs were significantly higher *in vitro* and *in vivo*. Thus, oat galactolipids could be used as a type of natural ligand of the ASGPR and a membrane material that would be beneficial for liver-targeting nanopreparations.

## 1 Introduction

Liver cancer is the sixth most common type of malignant tumor and the fourth most deadly type of cancer globally that is responsible for approximately 782,000 deaths each year (Sung et al., 2021; Ferlay et al., 2015). At present, despite the clinical availability of various anticancer strategies, including tumor immunotherapy, chemotherapy is still considered the conventional method for treating inoperable liver cancers. In general, chemotherapeutic agents, such as 5-fluorouracil, doxorubicin, and cantharidin, are considered “toxic” anticancer drugs that influence both malignant and normal cells. During chemotherapy, these drugs are distributed throughout the body because of lack of targeting and usually induce adverse effects (Hanaoka et al., 2015; Xu et al., 2016). Therefore, it is necessary to develop anticancer agents with lower toxicities and more effective liver-targeting drug delivery systems (LTDDSs).

Curcumin is a natural active metabolite isolated from the rhizomes of *Curcuma longa* and exhibits antitumor activities on liver, colon, gastric, and breast cancers, among others (Pan et al., 2018; Kronski et al., 2014; Wang et al., 2017; Toden et al., 2015). It has also been demonstrated to be safe in clinical research (Irving et al., 2013; Cheng et al., 2001) and is generally recognized as safe by the US Food and Drug Administration (Food and Drug Administration, 2011). Although curcumin has attracted wide attention as an antitumor natural metabolite with low toxicity, its preparation and clinical applications are limited because of its low water solubility, poor stability, and low bioavailability (Yallapu et al., 2015; Salem et al., 2014). To overcome these shortcomings, curcumin is usually formulated as a nanopreparation (Li et al., 2006; Mahmoudi et al., 2021).

Some studies have shown that nanopreparations modified by galactosyl ligands have liver-targeting capabilities as there are a large number of asialoglycoprotein receptors (ASGPRs) on the hepatocyte membranes. The ASGPR, also known as galactose receptor, is a kind of highly effective endocytosis receptor located on the surfaces of mammalian liver parenchymal cells (Sun et al., 2021) and overexpressed on the surfaces of hepatoma cells (D'Souza et al., 2015); it can specifically recognize galactose (gal) or acetylgalactosamine (galnac) residues at the ends of oligosaccharides or oligosaccharide proteins. In recent years, researchers have designed and synthesized a series of LTDDSs mediated by ASGPRs (Wang et al., 2020). However, in these reports of LTDDSs modified by galactosyl ligands, the ligands were prepared via chemical synthesis (Dong et al., 2017; Ding et al., 2018; Cheng et al., 2018; Li et al., 2018; Zhang et al., 2015; Liao et al., 2021) and expressed some shortcomings, such as multiple reaction steps, many byproducts, difficult separation and purification, solvent residue, and security issues.

In general, natural metabolites from food sources have greater biocompatibility and safety than synthetic materials. Studies have shown that plant galactolipids containing galactosyl are widely present in green plants (Andersson et al., 1997); however, there are no reports on liver-targeting nanocarriers modified with plant galactolipids and whether they have been investigated for liver targeting, which would be beneficial to the development of liver-targeting preparations. Galactolipids are mainly distributed in the chloroplast membranes of plants, and the main metabolites are monogalactodiglycerol (MGDG) and bisgalactoglyceride (DGDG). In 1956, Carter isolated MGDG and DGDG from wheat flour and determined their structures (Carter et al., 1956). Low concentrations of MGDG and DGDG have also been found to be present in the myelin sheaths and oligodendrocytes of animals (Schmidt-Schultz and Althaus, 1994). Therefore, galactolipids are natural metabolites of plants as well as physiological components of animals.


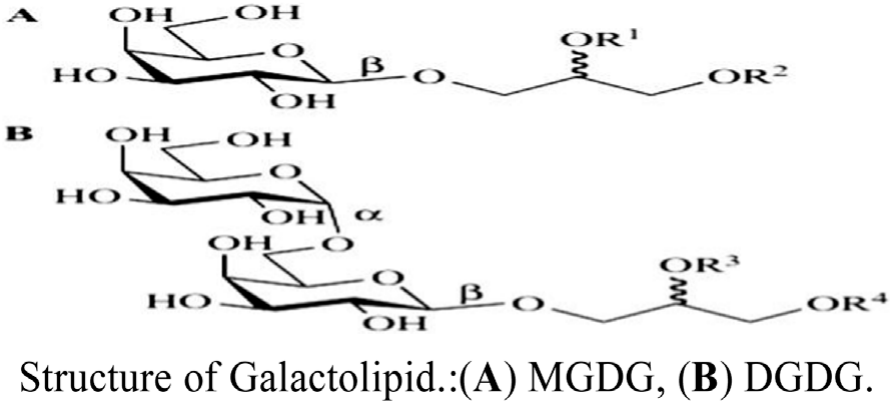


In a previous study, galactolipids were detected in oat, oat bran, wheat, rice, and other crop sources (Ren et al., 2022). Oat bran is a byproduct of oat processing and contains a high content of galactolipids, so we extracted these galactolipids from oat bran and prepared the oat-galactolipid-modified curcumin-loaded liver-targeting vesicles (GCLTVs) to investigate its liver-targeting and liver antitumor effects.

## 2 Materials and methods

### 2.1 Materials

Oat bran was purchased from Zhangjiakou Oat Food Development Co., Ltd. (Hebei, China). Curcumin was sourced from Hangzhou Sky Grass Technology Company Ltd. (Hangzhou, China). Cholesterol was obtained from Shanghai AVT Technology Pharmaceutical Ltd. (Shanghai, China). Tween-80 was procured from BioFroxx GmbH (Germany). RPMI-1640 culture medium and double resistance were sourced from Hyclone Company (United States). Mycoplasma-free fetal bovine serum (FBS) was obtained from Sijiqing Technology Co., Ltd. (China). Pancreatic enzymes were obtained from Gibco Company (United States). The Immuno–Bridge + kit used was from GBI Company (United States). The LDH-kit used was from Nanjing Jiancheng Bioengineering Institute (Nanjing, China). Anhydrous ethanol was procured from National Medicine Chemical Agent Co., Ltd. (China). The HepG2 (no. CL-0103) and H22 (no. CL-0341) cell lines were purchased from Procell Company (Wuhan, China). SPF-Kunming mice (male, 20 ± 2.0 g) were purchased from the Medical Animal Test Center of the Hubei Disease Control Center (Wuhan, China).

### 2.2 Preparation of oat galactolipids

The oat galactolipids were prepared in our laboratory according to our previous work (Ren et al., 2022). Oat bran was soaked in 85% ethanol for 30 min, extracted twice by ultrasonication for 30 min each time, and filtered, following which the combined filtrates were evaporated using a rotary evaporator; the remainder was dissolved in an appropriate amount of anhydrous ethanol and centrifuged to obtain the supernatant. Then, pure water was added to the supernatant, stirred, and maintained at 4°C for 12 h. The solution was filtered, the supernatant was evaporated by rotary evaporation, and the remainder was dissolved in an appropriate amount of anhydrous ethanol and left for a while before centrifugation to obtain the supernatant, following which the supernatant was evaporated to obtain the galactolipids (purity >96%).

### 2.3 Determination of acid value of galactolipids

One gram of the galactolipids was dissolved in a 50-mL mixture of ethanol and ether (1:1) in a 250-mL conical bottle, to which 1 mL of phenolphthalein indicator solution (0.1 g/mL) was added and titrated with NaOH solution (0.1 mol/mL) until the pink color persisted for 30 s. The volume (mL) of NaOH titrant consumed was denoted as A, and the weight (g) of the sample was denoted as W. The acid value was then calculated as follows:

Acid value=5.61×A/W.

### 2.4 Determination of the saponification value of galactolipids

The saponification value was detected according to a previous work (Chen et al., 2022). Briefly, 52.6 mg of the galactolipids was accurately weighed and placed in a 150-mL conical bottle, to which 25 mL of 0.5 M KOH ethanol solution was added, and the mixture was heated under reflux for 30 min. The condenser and stopper were then rinsed with 10 mL of ethanol, and 1.0 mL of phenolphthalein indicator was added and titrated with 0.5 M HCl until the pink color just disappeared. Then, the mixture was heated to boiling and titrated with HCl until the color faded. Here, the volume (mL) of HCl consumed was denoted as A, and the volume (mL) of HCl consumed in the blank test was denoted as B. The saponification value was then calculated as follows:

$$\text{Saponification value}=28.5\times \left(B-A\right)/W.$$

### 2.5 Determination of the ester value of galactolipids

Ester value=saponification value−acid value.

### 2.6 Determination of the hydrophilic–lipophilic binding (HLB) value of galactolipids

The HLB was detected according to a previous work (Luo et al., 2013). Briefly, the experimental temperature was to 40°C ± 1°C; then, 0.5 g of a mixture of standard samples of the surfactants Span-80 (A) and Tween-80 (B) with different proportions was weighed and introduced into a 100-mL conical flask, to which 5.8 mL of isopropanol and toluene were added as a mixed solution (volume ratio of 100:15) to prepare a surfactant solvent with a mass fraction of approximately 10%.

The number “3” was written on the bottom of the conical flask, which was placed on a white paper, and the flask was shaken with slow addition of distilled water with a burette until the number became blurred; here, the volume of distilled water consumed was recorded. The HLB value of the mixed surfactant was then calculated as follows:

$${\text{HLB}}_{0}=\left(W_{A}{\text{HLB}}_{A}+W_{B}{\text{HLB}}_{B}\right)/\left(W_{A}+W_{B}\right),$$

where HLB_0_ is the HLB value of the mixed surfactant, W_A_ is the mass of A, HLB_A_ is the HLB value of A, W_B_ is the mass of B, and HLB_B_ is the HLB value of B.

The standard curve of the HLB value was obtained, with HLB as the abscissa and volume of distilled water consumed as the ordinate. Then, 0.5 g of the mixed solution of galactolipids and Tween-80 was placed in a conical flask, and 5.8 mL of the mixed solution of isopropanol and toluene was added to prepare a surfactant organic solvent with a mass fraction of approximately 10%. The remaining procedures are the same as above. The HLB value of the galactolipids was then obtained from the standard curve.

### 2.7 Stability of the galactolipids

Three batches of galactolipid samples were prepared and stored at 4°C. Then, the acid, saponification, and ester values were each checked at 0, 3, and 6 months.

### 2.8 Preparation and prescription optimization of GCLTVs

In this study, cellular uptake was used as the judgment parameter for liver tumor cell targeting *in vitro* that revealed the liver tumor targeting effects to a certain extent and was used to optimize the formula of the GCLTVs. First, the basic formula of the GCLTVs was screened through orthogonal experiments, and the optimal ratio of galactolipids in the GCLTVs was selected based on the uptake by the HepG2 cells. The fluorescein isothiocyanate (FITC) value of cellular uptake was then detected according to the method outlined in Chang et al. (2018). Briefly, in a 50°C water bath, 150 mg of Tween-80, different proportions (15%–30% mass fraction) of galactolipids, 6 mg of cholesterol, and 4 mg of curcumin were dissolved in 1.5 mL of anhydrous ethanol and maintained for 10 min. Then, 1 mL of the residue solution was injected slowly into 20 mL of preheated (50°C) and stirred (250 rpm) distilled water. The solution was then stirred (250 rpm) at 50°C until the ethanol evaporated and was maintained at room temperature for 24 h; finally, the solution was filtered using a 220-nm microporous membrane to obtain 15%–30% GCLTVs.

The HepG2 cells (5 × 10^5^ cells/well) were seeded in 6-well plates with RPMI-1640 medium, followed by overnight incubation at 37°C and in an atmosphere containing 5% CO_2_; the medium was then removed, the cells were washed twice with phosphate-buffered saline (PBS), and the following treatments were applied: only RPMI-1640 medium (blank group); 10 μg/mL of free curcumin (curcumin group); 10 μg/mL of curcumin vesicles without galactolipids (curcumin vesicles group); 10 μg/mL of 10% galactolipid-modified curcumin vesicles (10% GCLTV group); 10 μg/mL of 15% galactolipid-modified curcumin vesicles (15% GCLTV group); 10 μg/mL of 20% galactolipid-modified curcumin vesicles (20% GCLTV group); 10 μg/mL of 25% galactolipid-modified curcumin vesicles (25% GCLTV group); 10 μg/mL of 30% galactolipid-modified curcumin vesicles (30% GCLTV group). After incubation for 2 h, the medium was removed, the cells were carefully washed twice with PBS, and the cells were digested with 0.25% trypsin without EDTA. Following digestion, the solution was centrifuged (5 min at 1500 rpm) to harvest the HepG2 cells, which were then resuspended in 500 μL of precooled PBS. The uptake of curcumin was measured via flow cytometry (BD Biosciences, United States) at an excitation wavelength of 488 nm and emission wavelength of 530 nm. The maximum uptake group was selected as the optimal prescription of GCLTVs, which was then used in the follow-up experiments.

### 2.9 Entrapment efficiency (EE) and content of GCLTVs

An appropriate amount of GAMCLCL was diluted 30 times with double-distilled water, and the morphology of the GCLTVs was detected via transmission electron microscopy (Japan). The particle size and potential were then determined using a ZS90 nanoparticle instrument (Malvern, United Kingdom). Three batches of GCLTVs were prepared, and their curcumin contents were determined by high-performance liquid chromatography (HPLC) (Wen-ru et al., 2022). The chromatographic conditions used were as follows: Boston Green ODS C-18 column (250 mm × 4.6 mm, 5 μm); mobile phase containing methanol at 0.1% and acetic acid in water (80:20); flow rate of 1.0 mL/min; detection wavelength of 429 nm; sample injection volume of 10 μL. The curcumin standard solution was prepared as follows: approximately 10 mg of curcumin standard was weighed into a 10-mL volumetric flask, the volume was adjusted to scale with methanol, and 1 mg/mL of the standard solution was prepared. Then, the standard curve was obtained and the methodology was investigated. The curcumin contents were calculated from the standard curve.

The EE of the GCLTVs was determined according to a previous work (Lv et al., 2022). Briefly, 2 mL of the vesicle solution stored at 4°C for 24 h was centrifuged at 3,000 rpm for 10 min. The curcumin is insoluble in water and is thus precipitated. A 0.10-μm microporous membrane was used to remove the unencapsulated drugs. Then, 1 mL of the filtrate was placed in a 10-mL volumetric flask, to which methanol was added, vortexed, mixed well, ultrasonically demulsified, and shaken well until the volume reached the scale. The sample was then detected by HPLC, which was recorded as m_1_, and the dosage of curcumin in the formula was recorded as m_2_. The EE was then calculated as

$$\text{EE}=m_{1}/m_{2}\times 100\%.$$

### 2.10 Morphological stability of the GCLTVs

Artificial gastric juice was prepared by adding 16.4 mL of dilute hydrochloric acid to 800 mL of water along with 10 g of pepsin and shaken well; then, water was added till the volume was 1,000 mL. Artificial intestinal fluid was prepared by dissolving 6.8 g of potassium dihydrogen phosphate in 500 mL of water and adjusting the pH to 6.8 with 0.1 mol/L of sodium hydroxide solution; then, 10 g of pancreatin was dissolved in an appropriate amount of water and the two fluids were mixed and diluted to a volume of 1,000 mL. The GCLTV solution was mixed with the artificial gastric juice and artificial intestinal fluid in a 1:1 ratio, and the appearance, morphological changes, particle size, and potential were observed after maintaining in a water bath at 37°C for 48 h.

### 2.11 Release of GCLTVs *in vitro*

The GCLTV solution was added to a dialysis bag, clamped at both ends, placed in 50 mL of a release medium (artificial gastric juice or artificial intestinal fluid containing 30% ethanol), incubated at (37 ± 2)°C, protected from light, and incubated further in a water bath at constant temperature. Then, 5.0 mL of the dialysate was aspirated at 0.5, 1, 2, 3, 4, 5, 6, 8, 10, 12, and 24 h, and 5.0 mL of a blank release medium was added at the same temperature. The dialysate was then filtered using a 0.22-μm microporous membrane, and the drug concentration was determined by HPLC.

### 2.12 Cellular uptake of GCLTVs

The cellular uptake of GCLTVs was performed as per the above experiments as part of the prescription optimization of GCLTVs. The treated groups were as follows: 10 μg/mL of curcumin, 10 μg/mL of curcumin vesicles, and 10 μg/mL of 30% GCLTVs. The treatment times were 1, 2, and 4 h each. Then, the FITC values were detected via flow cytometry.

### 2.13 Study on liver targeting *in vivo*

Three mice were selected as the blank control group using a randomized numeric table method, and 24 mice were randomly divided into two the curcumin vesicles and GCLTV groups. After the mice were fasted without water for 12 h, each group of mice was administered 40 mg/kg of each of the treatments by gavage. After collecting blood, three mice were sacrificed at the 0.5, 1, 2, and 4 h points; their livers were collected, and the blood was dried using filter paper. We then weighed 0.1 g of each liver sample, added 1 mL of the solvent (ethyl acetate and methanol in the ratio of 9:1), homogenized at 30 Hz in a high-speed tissue homogenizer for 1 min, and repeated the process twice more. The homogenates were centrifuged at 10,000 rpm for 10 min, supernatants were removed and evaporated in a water bath at 50°C, residues were dissolved in methanol and filtered through 0.1-μm micropores, and curcumin contents were determined by HPLC.

The liver drug-time curve in the form of area under the curve (AUC) was plotted based on the mean drug concentration at each time point. The liver targeting of GLCTVs was evaluated using the targeting efficiency (te) and peak concentration ratio (Ce) metrics, whose calculation formulas are as follows:

te=AUC1/AUC2.

Ce=C1/C2.

Here, AUC1 represents the area under the curve of the GLCTVs, and AUC2 represents the area under the curve of the curcumin vesicle group; C1 represents the peak concentration of the GLCTVs, and C2 represents the peak concentration of the curcumin vesicle group. When Ce and te were greater than 1, the GCLTV preparation showed better targeting efficacy.

### 2.14 Cytotoxicity assay on the HepG2 cells

The cytotoxicity assay was conducted in accordance with an earlier work (Chang et al., 2020). The HepG2 cells (5,000/well) were seeded in 96-well plates with 100 mL/well of the medium and incubated overnight with 10% FBS, RPMI-1640 medium at 37°C, and an atmosphere of 5% CO_2_. The curcumin, curcumin vesicles, and GCLTVs were added to the HepG2 cells at various concentrations, where the concentrations of curcumin in the three kinds of drugs were varied as 5, 10, 15, 20, and 40 μg/mL. After treatment for 24 h and 48 h, the medium was removed and RPMI-1640 medium containing 10% CCK-8 was added. After incubation for 30 min at 37°C, the absorbance (A) at 450 nm was measured using an automatic enzyme standard instrument (Multiskan MK3, USA). The inhibition rate (IR) of cellular proliferation was calculated as follows:

$$\text{IR}=\left(1-A\text{ experimental group}\right)/\;A\text{ control group}\times 100\%.$$

### 2.15 Orthotopic xenograft model of liver and administration methods

The orthotopic xenograft model was prepared according to an earlier work (Jie et al., 2015). Briefly, the ascites fluid samples of mice inoculated with H22 hepatoma cells were extracted, and PBS was added. The cell counts were then obtained after centrifugation, and the cell density was adjusted to 2 × 10^7^ cells/mL using normal saline. Then, 24 mice were fed for 1 week and randomly divided into four groups with six animals in each group. These were designated as the model group, model group + free curcumin group, model group + GCLTV group, and model group + curcumin vesicles group. After fasting for 12 h, the mice were shaved on the abdomen, anesthetized, and fixed on a mouse plate in a supine position. The abdomen was disinfected with 0.5% iodine, and an incision of approximately 1.0 cm was made. The abdomen was then cut along the white line, separated layer by layer, and hemostasis was performed. The bilateral abdominal walls of the mice were gently squeezed such that the liver bulged out of the upper abdomen. The prepared H22 cell suspension was then inserted into the liver lobe at a depth of approximately 0.1 cm, and the cancerous ascites were slowly pushed. Using a multipoint injection approach, 10–20 μL of the cell liquid was injected at each point for a total volume of 50 μL. The needle was then removed quickly and the area was disinfected using a cotton swab pressed over the puncture point until there was no bleeding; then, a burning red wire was used to lightly ignite the needle eye to close the needle aperture to prevent tumor cells from escaping. Then, the liver was reinserted, and the abdominal cavity was closed layer by layer. The wound was finally disinfected with erythromycin ointment. One week later, the mice were orally administered saline and three kinds of drugs once a day at 40 mg/kg; after 1 week of continuous administration, the blood samples were obtained and tested for liver function (ALT, AST). Then, the mice were anesthetized and sacrificed by neck-breaking, and the livers of the mice were dissected to observe the growth of orthotopic liver tumors for the subsequent tests.

### 2.16 H&E staining

Liver tissues containing tumors were selected and fixed with 4% paraformaldehyde, stained with H&E and observed under an optical microscope.

### 2.17 Western blotting

The Western blot method was used to detect the expressions of p53, Bax, and Bcl-2 in the tumor masses according to earlier works (Xu et al., 2022;Zhang et al., 2021). A small piece of the tumor tissue was collected from each mouse, grounded, cracked, and centrifuged. The protein concentration of each diluted sample was determined using the BCA assay kit. The total protein (40 μg) and markers were resolved by electrophoresis and transferred to polyvinylidene fluoride (PVDF) membranes. These membranes were blocked in TBST containing 5% skimmed milk for 2 h at room temperature and then incubated overnight at 4°C with the antibodies of P53, Bax, and Bcl-2. After five full washes in TBST, the membranes were incubated with anti-mouse IgG antibody labeled with horseradish peroxidase for 2 h. The membranes were then exposed to X-ray film before being rinsed, dried, and scanned; then, the gray values of the films were computed using BandScan software.

### 2.18 Statistical analysis

All quantitative data were reported as mean ± standard deviation (SD). A *t*-test was used to test the differences between the groups. The significance level was set at *p* < 0.05, and *p* < 0.01 indicated an extremely significant difference.

## 3 Results

### 3.1 Properties of galactolipids

**FIGURE 1:**
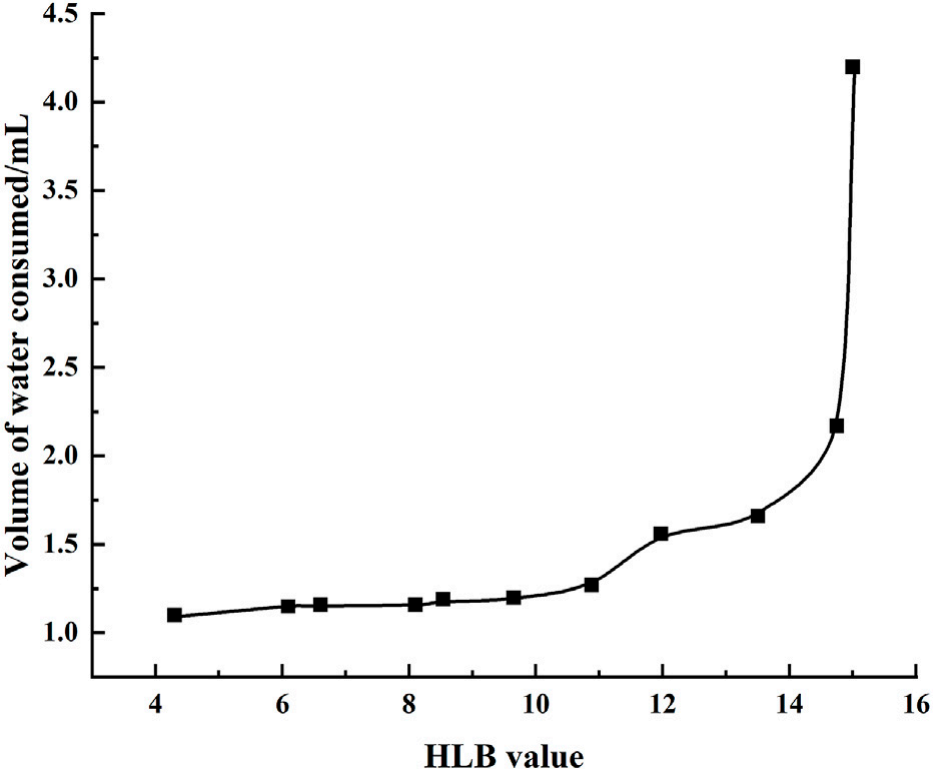
HLB value curve.

Based on the preparation process outlined herein, the yield of galactolipids extracted from oat bran was 569.61 ± 1.62 ( mg/100 g), RSD = =0.28% (n = 3). Its appearance was an orange-yellow grease, and the following metrics were noted: HLB value (Figure 1) of 14.89 ± 0.06 (n=3); acid value of 47.22 ± 0.94 (n=3); ester value of 237.09 ± 2.89 (n=3); saponification value was of 284.30 ± 2.03 (n=3). The extracting extraction results of the three batches are shown in Table 1. During the 6 months of observations, the acid, ester, and saponification values of the galactolipids remained stable (Table 2).

**TABLE 1:** Extraction results of the three batches (mg/100 g) (n = 3).

Batch	1	2	3	Mean	SD
Result	0.564	0.572	0.552	0.563	0.008

**TABLE 2:** Stability of the prepared galactolipids (months) (n = 3).

	0	3	6
Acid value	47.22 ± 0.94	45.54 ± 0.54	47.19 ± 0.23
Ester value	237.09 ± 2.89	236.05 ± 3.29	227.27 ± 2.24
Saponification value	284.30 ± 2.03	281.59 ± 2.88	274.46 ± 2.43

### 3.2 Optimal prescription of GCLTVs

**FIGURE 2:**
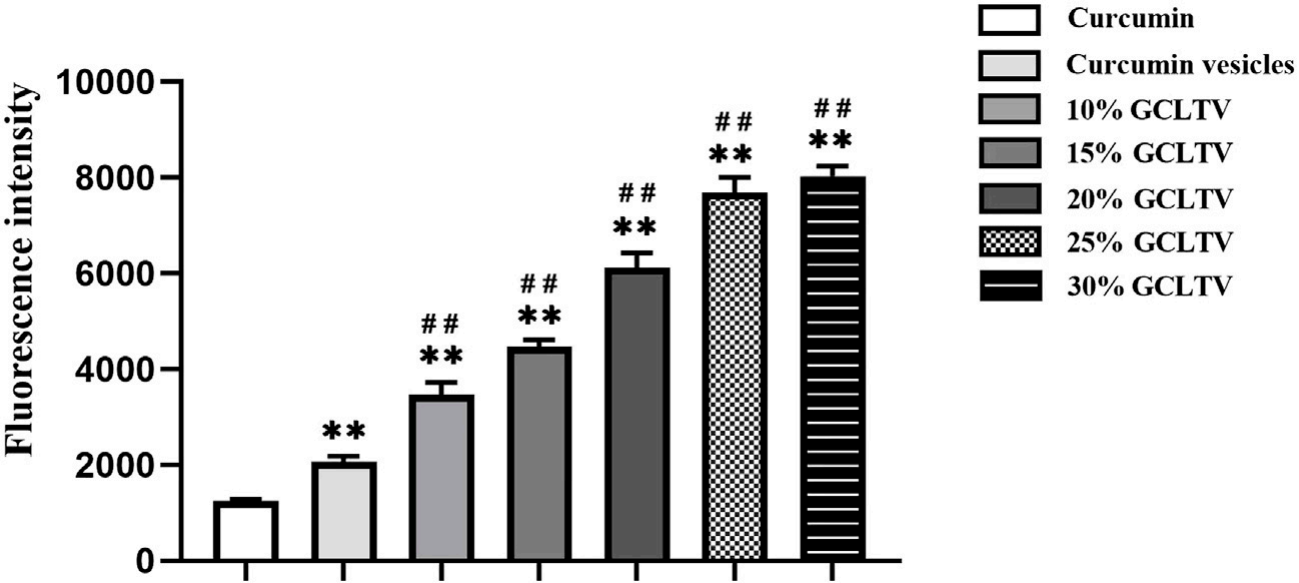
Cellular uptakes of groups with different proportions of active components. The FITC values of curcumin in the different groups were detected by flow cytometry. The uptake intensities were positively correlated with the proportions of galactolipids accounting for 10%–30% of GCLTVs. Compared with the free curcumin group, **p < 0.01. Compared with the curcumin vesicle group, ^##^ *p* < 0.01 (n = 3).

Previous experiments have shown that GCLTVs would be unstable in artificial gastric juice when the galactolipids account for 50%. The stability of the preparation *in vivo* is key to the preparation research, so we chose 0%–30% proportion of galactolipids as the research scope to ensure the stabilities of the preparations. The uptake results of different galactolipid proportions are shown in Figure 2. The cellular uptakes of different galactolipid proportions show that compared with the curcumin vesicle group, the uptake intensities are positively correlated with the proportion of galactolipids in the 10%–30% range of GCLTVs. Although the uptake intensity at 30% was higher than that at 25%, there was no significant increase, so the optimal galactolipid proportion was selected as 30% for the subsequent tests.

### 3.3 Characteristics and stabilities of the GCLTVs

**FIGURE 3:**
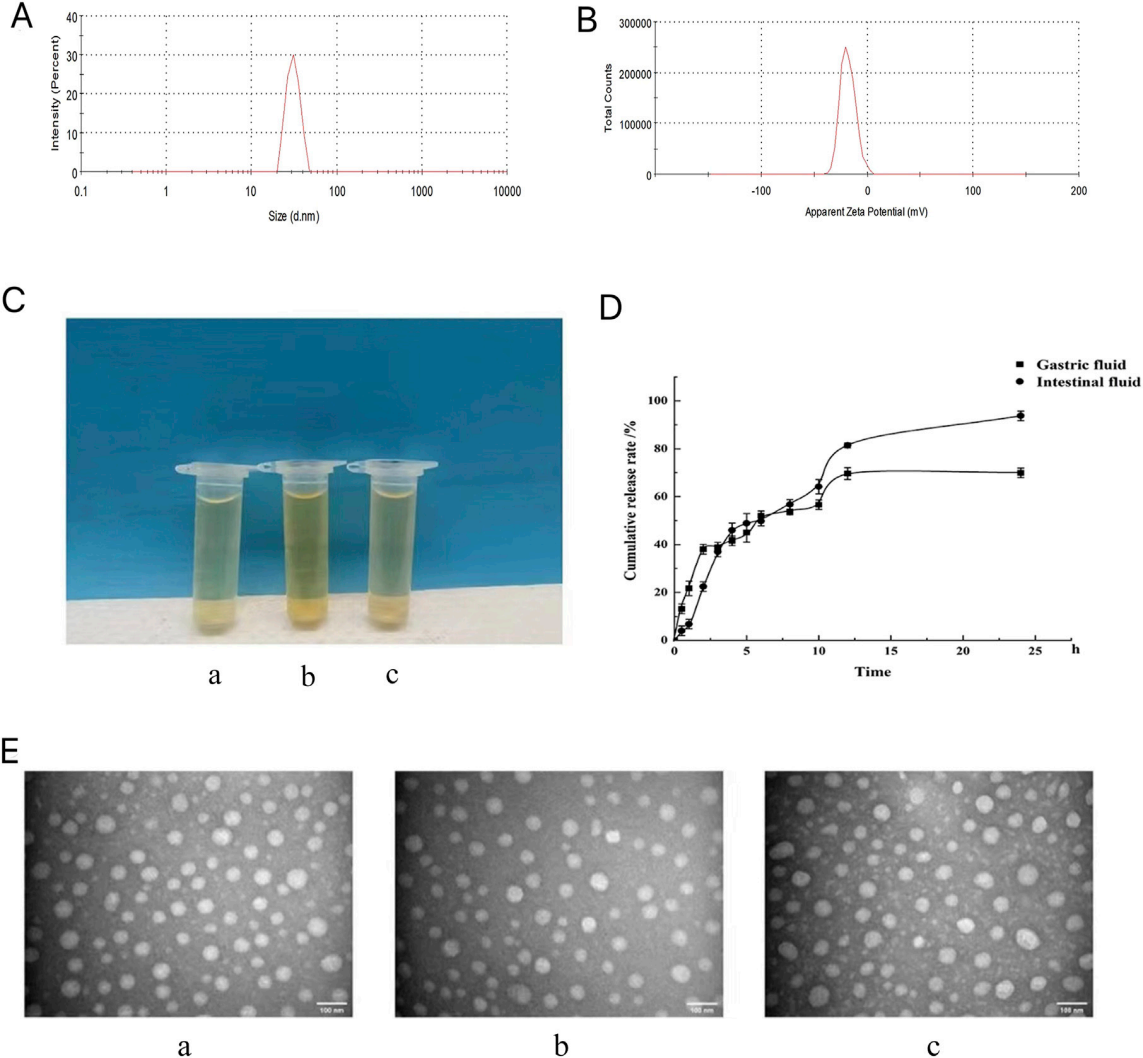
Microscopic characteristics, stability, and release results of the GCLTVs: **(A)** particle size distribution of the GCLTVs; **(B)** measured potential of the GCLTVs; **(C)** appearances of the three transparent vesicle solutions (a. GCLTVs + artificial gastric juice, b. GCLTVs, and c. GCLTVs + artificial intestinal fluid); **(D)** release of GCLTVs in the artificial gastric juice and artificial intestinal fluid; **(E)** transmission electron microscopy images of the three kinds of GCLTV solutions showing similar particle sizes (a. GCLTVs + artificial gastric juice, b. GCLTVs, c. GCLTVs + artificial intestinal fluid).

The morphology of the GCLTV is shown in Figures 3B–E. The vesicle has a spherical appearance, with an average particle size of 64.47 ± 0.67 nm (Figure 3A) and an average potential of −19.73 ± 1.08 mV (n = 3) (Figure 3B). The stability results show that GCLTVs could remain stable in the gastrointestinal fluid for 48 h. The three solutions were transparent, and there was no precipitation (as shown in Figure 3C). The three vesicle samples has spherical shapes under electron microscope, as shown in Figure 3E. The average particle sizes were 63.55 ± 0.68 nm in artificial gastric juice, 66.4 ± 1.41 nm under normal conditions, and 69.5 ± 1.37 nm in artificial intestinal fluid. The EE of the GCLTVs was $92.06\pm 0.0.43\%\;\left(n=3\right)$ . The curcumin contents of the GCLTVs are shown in Table 3.

**TABLE 3:** Curcumin contents of the three batches (μg/mL) (n = 3).

Batch	1	2	3	Mean	SD
Result	182.29	182.58	176.46	180.44	2.82

### 3.4 Release results of GCLTVs

The release results (Figure 3D) show that the GCLTV solution releases up to 70% of the drug in artificial gastric juice within 24 h and 93% of the drug in artificial intestinal fluid within 24 h.

### 3.5 Tumor cell targeting *in vitro*

**FIGURE 4:**
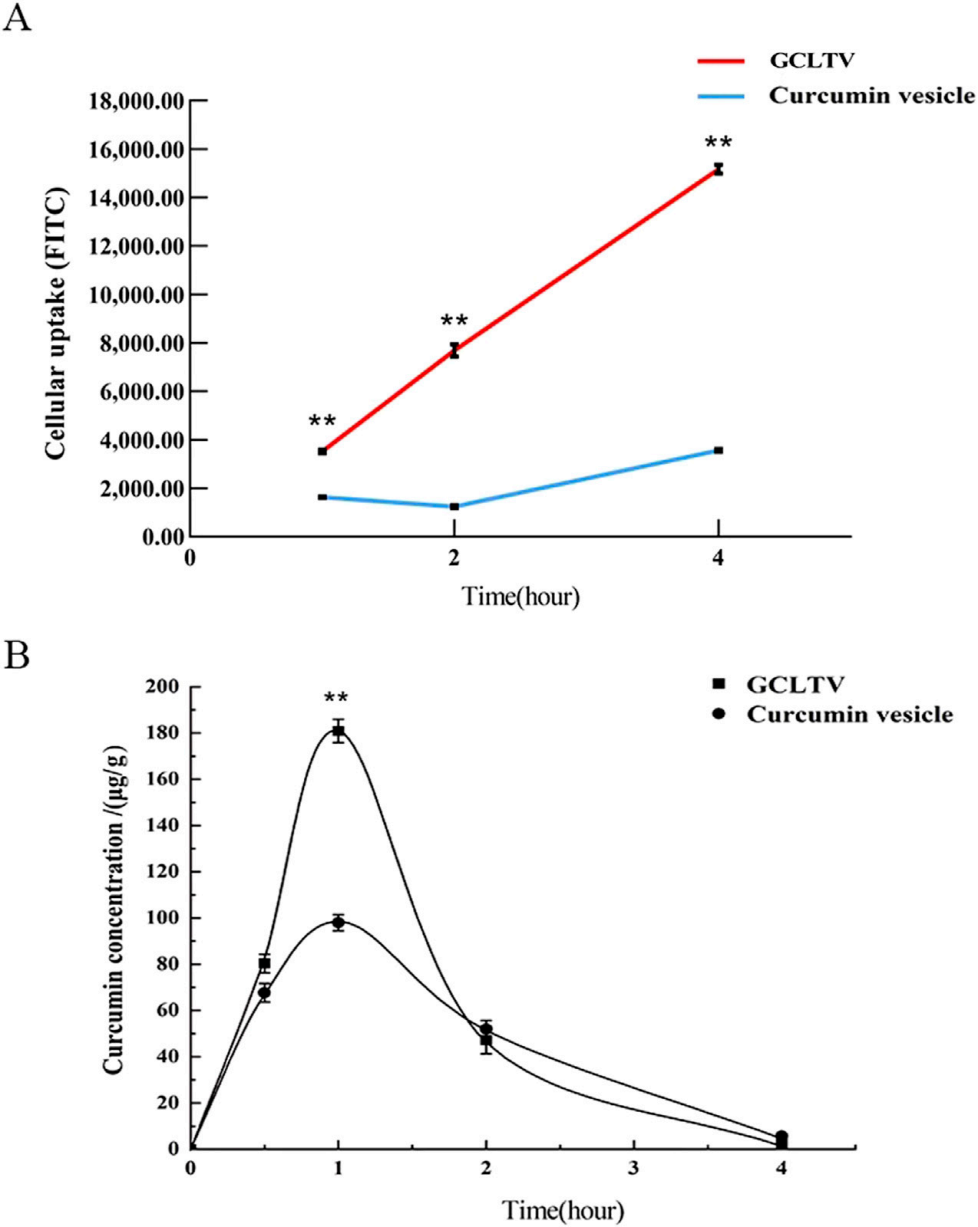
Targeting effects *in vitro* and *in vivo* (n = 3). **(A)** Line chart of cellular uptake at different times. The FITC values of curcumin uptake by the HepG2 cells were detected by flow cytometry. **(B)** Drug concentration in the liver versus time curve. The drug concentration in the liver was highest at 1 h, and the peak concentration of the GCLTV group was 1.85 times that of the curcumin vesicle group. Compared with curcumin vesicle group, **p < 0.01.

To investigate the targeting of GCLTVs in liver tumor cells, the HepG2 cellular uptakes were assessed at different times. These uptake results are shown in Figure 4A. The results show that compared with the curcumin vesicles without galactolipid modification, the uptake of GCLTVs was significantly higher at the same time point. At 1 h, the uptake intensity of GCLTVs was twice that in the curcumin vesicle group, four times at 2 h, and five times at 4 h.

### 3.6 Liver targeting of GCLTVs *in vivo*

The contents of curcumin in the samples were determined by HPLC, and the regression equation of the standard curve is as follows:

$$Y=2216.3x+26.455\;\left(R=0.999,n=6\right).$$

There was a good linear relationship between the curcumin content (y) and peak area (x) in the concentration range of 0.01–0.50 mg/mL. The test solutions were stable within 10 h, and the recovery rate was 102.26%, RSD = 0.02%. The drug-time curves of the mice livers at different time points are shown in Figure 4B. The maximum concentrations of GCLTVs and curcumin vesicles in the liver were 180.88 ± 3.57 μg/mL and 98.01 ± 2.65 μg/mL, and the AUCs of the GCLTVs and curcumin vesicles were 309.6 μg/mL and 220.5 μg/mL∙min over 240 min, respectively, which show that all the results of the GCLTV group are significantly higher than those of the curcumin vesicle group. The liver-targeting efficiency (te) was 1.40 (>1) within 240 min, and the peak concentration ratio (Ce) was 1.85 (>1), indicating that galactolipid modification significantly improves the effects of the GCLTVs.

### 3.7 Inhibitory effects of GCLTVs on HepG2 cells

**FIGURE 5:**
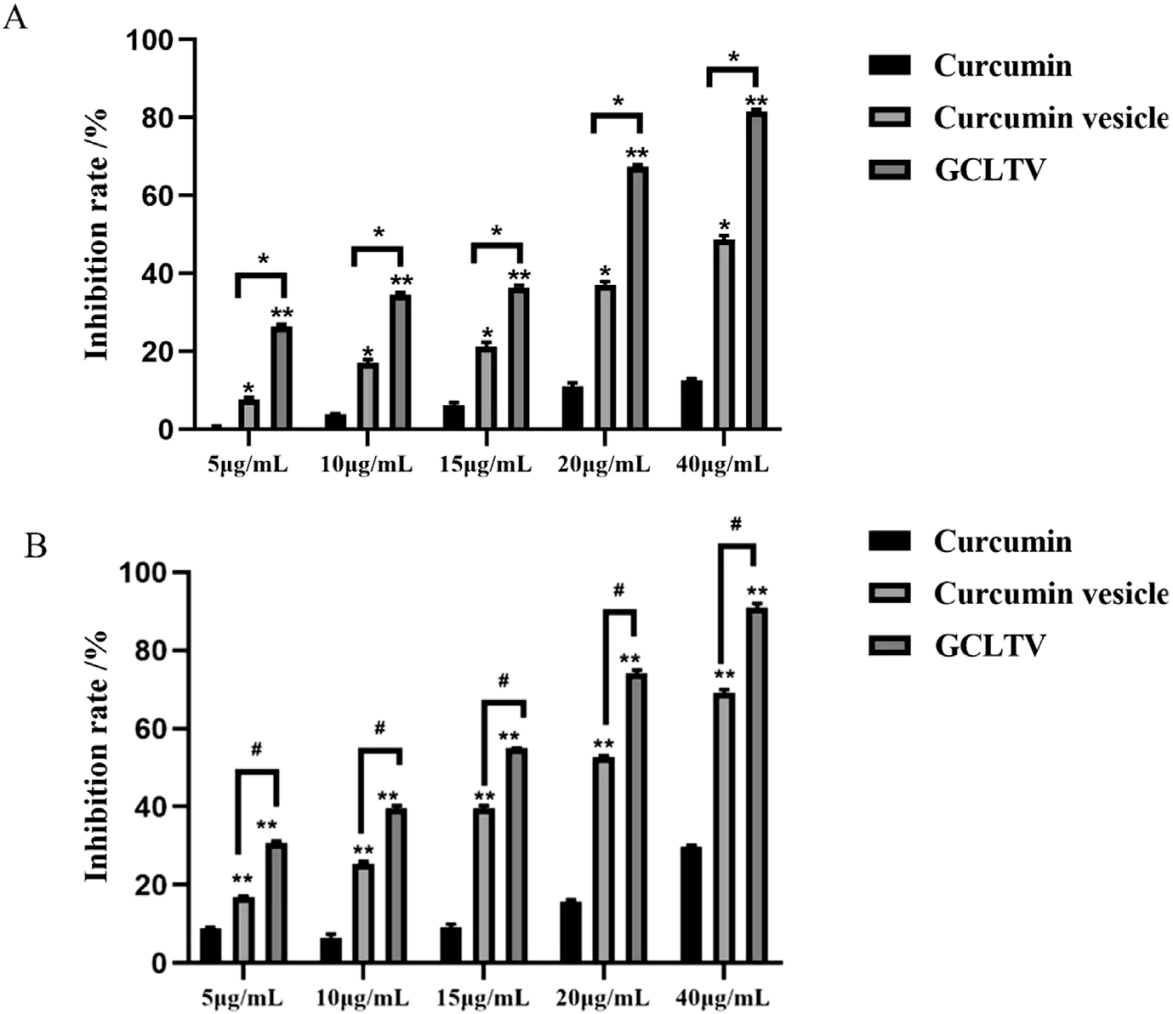
Bar chart of the inhibitory effects on the HepG2 cells (n = 5). **(A)** Inhibitory effects for different concentrations of different drugs at 24 h. **(B)** Inhibitory effects for different concentrations of different drugs at 48 h (*p < 0.05, **p < 0.01).

As shown in Figure 5, free curcumin had weak inhibitory effects on the proliferation of HepG2 cells. Compared with free curcumin, the inhibitory effects of GCLTVs and curcumin-loaded vesicles were higher at different concentrations at the ends of 24 h and 48 h (*p* < 0.01), and the inhibitory effects of GCLTVs were significantly higher than those of the curcumin vesicles (*p* < 0.05).

### 3.8 Antitumor effects of GCLTVs *in vivo*

**FIGURE 6:**
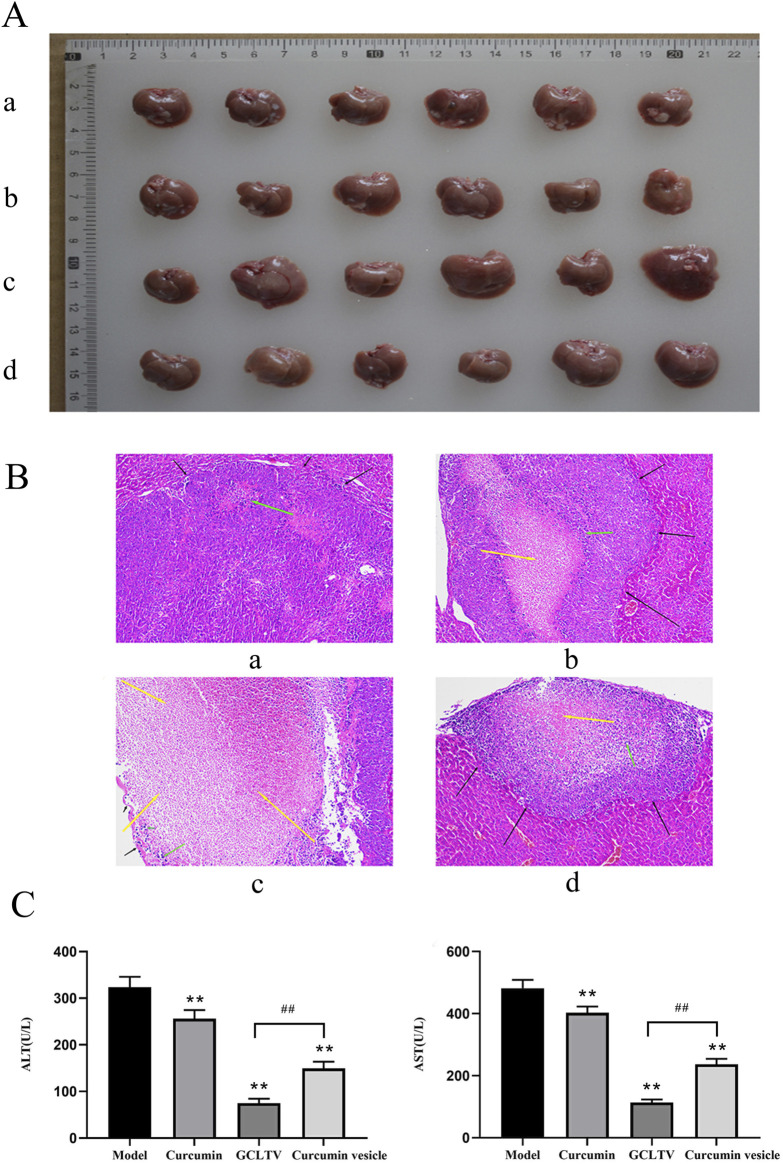
Antitumor effects of GCLTVs (n = 6). **(A)** Livers and tumors of the different groups (a. model group, b. GCLTV group, c. curcumin vesicle group, and d. free curcumin group). **(B)** H&E staining results for the different groups (a. model group, b. GCLTV group, c. curcumin vesicle group, and d. free curcumin group). **(C)** Bar chart of ALT and AST levels. Compared with the model group, **p < 0.01; compared with the curcumin vesicle group, ^##^ *p* < 0.01.

The liver and tumor samples of all the treatment groups are shown in Figure 6A. Since the H22 cell line was inoculated in the mouse liver through point injection, the corresponding tumors show white plaques. In the model group, the lesions were gray and hard and prominently present on the surface of the liver. The H&E stained images are shown in Figure 6B. The black arrows in these images refer to the tumors, yellow arrows indicate the necrotic areas of the tumors, and green arrows are the inflammatory cells. The tissue morphology in the model group was complete with obvious tumors in the liver tissue; further, the boundary was clear, and there was a small amount of inflammatory cell infiltration in the tumor, which showed that the model was successful. The tissue morphology of the curcumin group was intact, with obvious tumor formation, a small amount of inflammatory cell infiltration, and a small area of necrosis in the tumor. The tissue morphology and structure of the curcumin vesicle group were complete, and there were obvious tumors in the liver tissue with clear boundaries; medium-sized necrotic areas were present in the tumors, accompanied by small amounts of inflammatory cell infiltration. The histological structure of the GCLTV group was complete, with a large area of necrosis in the tumor accompanied by inflammatory cell infiltration.

**FIGURE 7:**
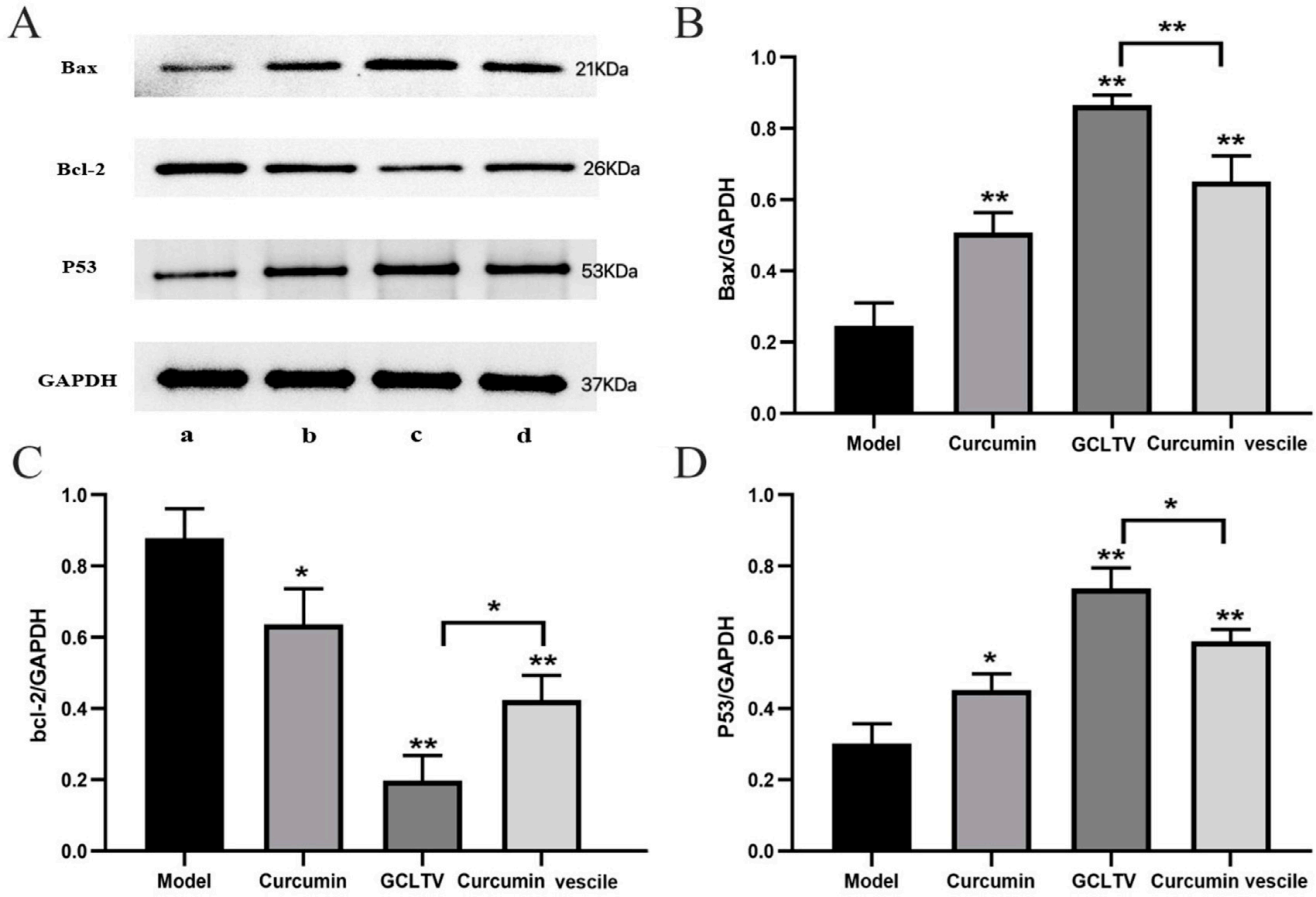
Results of the Western blotting test (n = 6). **(A)** Western blot pictures of Bax, Bcl-2, P53, and GAPDH in the different groups with molecular weights of 21, 26, 53, and 37 kDa, respectively (a. model group, b. GCLTV group, c. curcumin vesicle group, and d. free curcumin group). **(B)** Bar chart of Bax expressions in different groups, where GCLTVs upregulate Bax expression. **(C)** Bar chart of Bcl-2 expressions in different groups, where GCLTVs downregulate Bcl-2 expression. **(D)** Bar chart of P53 expressions in different groups, where GCLTVs upregulate P53 expression. Compared with the model group, *p < 0.05; **p < 0.01; compared with the curcumin vesicle group, ^#^ *p* < 0.05; ^##^ *p* < 0.01.

According to the H&E staining results, the curcumin group showed weak effects on the liver tumors, while the curcumin vesicles and GCLTVs showed stronger effects, with the GCLTVs showing stronger effects than the curcumin vesicles. The liver function results are shown in Figure 6C. The results show that compared with the free curcumin group, the ALT and AST values of the curcumin vesicle and GCLTV groups were significantly lower and that the effects of GCLTVs on the ALT and AST levels were more significant. The Western blotting results are shown in Figure 7. Accordingly, compared with the free curcumin group, the expressions of P53 and Bax in the curcumin vesicle and GCLTV groups were significantly higher, expressions of Bcl-2 were significantly lower, and effects of GCLTVs on P53, Bcl-2, and Bax were more significant.

## 4 Discussion

LTDDSs can efficiently convey drugs to the liver and increase the drug efficacy. When preparing the LTDDS, the membrane material and ligand were the key accessories that affected the stability, EE, and targeting efficiency of the nanopreparation. Because the receptors are usually bound to small molecules or groups and the ligands modifying the LTDDSs must be embedded in them, the small molecules or groups cannot be used as the ligands of LTDDSs directly; hence, the ligands are usually prepared by chemical syntheses of these small molecules or groups with cholesterol or other compounds, and there are no reports on the direct use of natural ligands in LTDDSs.

The molecular structures of plant galactolipids are similar to lecithin; these galactolipids have galactosyl ligands, surface activity, and film-forming characteristics along with the ability to be embedded in LTDDS, so they can be used as natural ligands of ASGPRs to avoid shortcomings in the chemical synthesis of the ligands. Until now, there have been no reports on liver-targeting nanopreparations modified with plant galactolipids. Presently, plant galactolipids are mainly extracted by sectional elution and separation of silica gel columns with different proportions of organic solvents (such as chloroform:methanol) (Woźniak et al., 2021; Akasaka et al., 2016). The extraction methods have some limitations, such as low yield, high cost, and residues of the organic solvents in the products. Hence, we developed a new extraction process to prepare oat galactolipids from oat bran using ethanol and water as the solvents instead of chloroform and methanol, such that the process incurs low cost, has good security, and achieves high purity.

The fatty acids present in the side chains of the galactolipids are mainly oleic acid, linoleic acid, linolenic acid, palmitic acid, stearic acid, etc. Thus, the characteristics were determined using the acid, saponification, and HLB values. The results show that the HLB value of oat galactolipids is 14.89, which is obviously higher than the 9–12 of lecithin that is commonly used as the membrane material for nanopreparations, indicating that oat galactolipids possess excellent surface activity and emulsifying ability. Therefore, when preparing liver-targeting nanopreparations modified with oat galactolipids, the galactolipids can be used as both ligands and membrane materials, and the preparation process would be greatly simplified in addition to improving the druggability and stability of the nanopreparation. In this study, GCLTVs were successfully prepared by the ethanol injection method under mild conditions.

During nanopreparation targeting studies, free drugs are usually adopted as controls, but the low bioavailability of curcumin presents difficulty in detecting its concentration *in vivo* upon oral administration of free curcumin as the control; hence, curcumin-loaded vesicles without oat galactolipids were used as the control *in vitro* and *in vivo*. If curcumin was directly used as the control, the difference in the results between the two groups would be more significant.

The formulation of a liver-targeting preparation is closely related to its effects, and the ligand proportion in the formulation is an important parameter of the nanopreparation. Screening prescriptions for targeting through direct animal tests would involve a heavy workload. In the preparation of GCLTVs, an orthogonal design approach combined with cell absorption was used to optimize the formula, which avoided problems like the orthogonal experimental results not guaranteeing the absorption effects of liver cancer cells and the cell absorption experiments not guaranteeing the stability of the preparation. The results of the liver-targeting experiments *in vivo* showed that the AUC in GCLTV group was much higher than that of the curcumin vesicle group within 4 h, indicating that the distribution of curcumin in the liver tissue could be significantly improved after modification with galactolipids. For the GCLTVs, the liver-targeting efficiency (te) was 1.40 (>1) and peak concentration ratio (Ce) was 1.85 (>1), indicating that the oat galactolipid modification significantly increased the curcumin concentration from the GCLTVs in the liver.

Additionally, a literature review showed that the targeting studies of nanopreparations were mostly carried out through intravenous injection and that such studies have not been reported for oral administration. The treatment of liver cancer requires long-term administration, for which interventional injections can cause injury, inconvenience, and compliance problems to the patients; in such cases, oral administration is the most preferred mode for patients as it is convenient to imbibe and carry. Our study indicates that oral administration of GCLTVs could obviously increase the concentration of curcumin delivered to the liver, thereby showing excellent liver targeting and providing a reference for the oral targeting studies of nanopreparations in the future.

The results of cell proliferation inhibition show that compared with the curcumin-loaded vesicles, the inhibitory effects of GCLTVs are significantly higher as well as time- and concentration-dependent, implying that GCLTVs have better antitumor effects. The *in vivo* results of H&E staining, liver function tests, and Western blotting analysis show that the antitumor effects are significantly improved after administration of GCLTVs compared with curcumin vesicles.

Apoptosis is considered to be an important anticancer effect. In the process of inducing apoptosis, the P53 and Bcl-2 families play different roles, and their abnormal functions are related to many cancers and autoimmune diseases. P53 is a tumor suppressor gene that regulates various metabolic pathways and maintains cellular metabolic homeostasis to induce apoptosis (Hao et al., 2023). Bcl-2 plays a role in inhibiting apoptosis as its high expression increases tumor cell proliferation. Bax plays an antiapoptotic role, where its high expression induces apoptosis of tumor cells and inhibits tumor growth (Zheng et al., 2020). The results show that the expressions of P53 and Bax are significantly increased and that of Bcl-2 is significantly decreased after administration of curcumin vesicles and GCLTVs, with the effects of GCLTVs on P53, Bcl-2, and Bax being more significant. Therefore, curcumin vesicles modified with galactolipids could obviously promote tumor apoptosis by upregulating P53 and Bax expressions while downregulating Bcl-2 expression. The reason for this is that the concentration of curcumin in the liver tumor increases after modification with galactolipids, thus increasing the effects of the GCLTVs in regulating P53, Bcl-2, and Bax levels.

In conclusion, oat galactolipids were prepared from oat bran through a new extraction process involving galactosyl ligands with excellent security and emulsifying ability. The experimental results show that GCLTVs have excellent liver-targeting abilities *in vitro* and *in vivo* while exhibiting much better antitumor effects than curcumin vesicles *in vitro* and *in vivo*. Therefore, oat galactolipids could be developed into a kind of natural ligand for ASGPRs and as nanopreparation membrane materials that would be beneficial for the formulation of liver-targeting preparations. The GCLTVs demonstrated in this work can be administered orally, which would reduce the preparation cost while increasing the convenience and compliance of medication, thereby making them worthy objects of further development and research.

## Data Availability

The original contributions presented in this study are included in the article/supplementary material, and any further inquiries may be directed to the corresponding authors.
