# Supplementary figures and images for: Preparation of oat galactolipid and anti-liver cancer effects of oat galactolipid–modified curcumin-loaded liver targeting vesicle
Source: Front Pharmacol. 2025 Jan 8;15:1511666. doi: 10.3389/fphar.2024.1511666 (PMC11751016; doi:10.3389/fphar.2024.1511666)

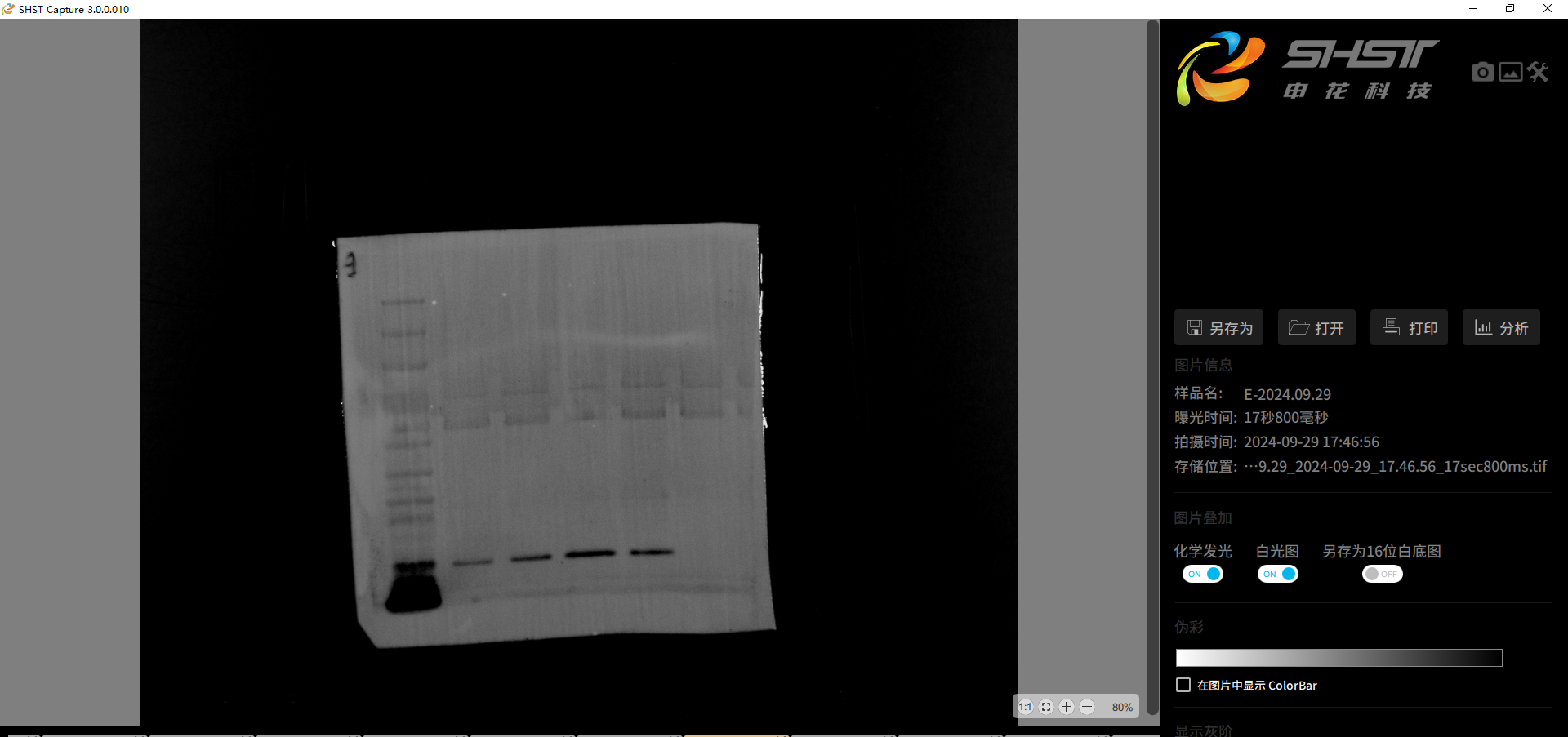

Supplement: Supplementary file 1 [file DataSheet1.zip › Original data-WB/bax-Original data.JPG]

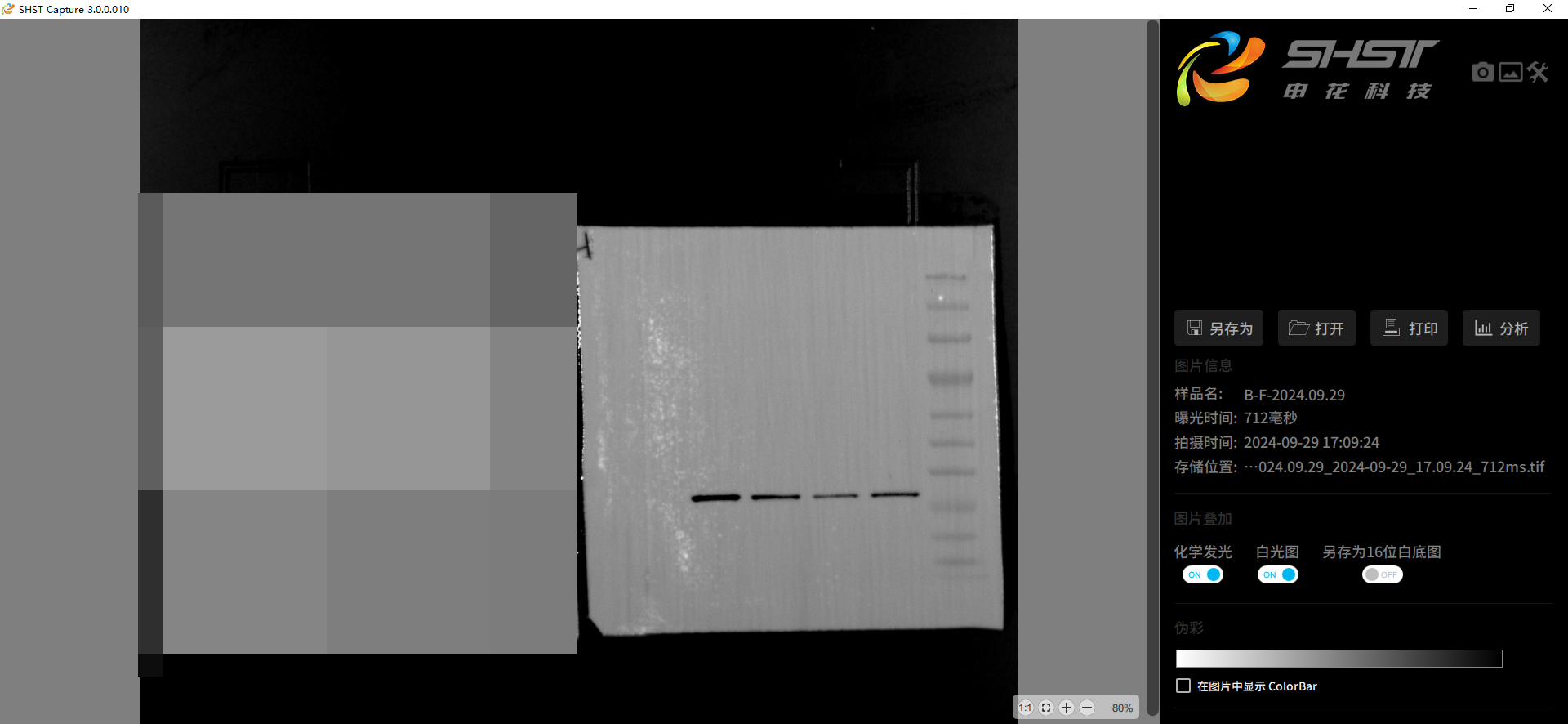

Supplement: Supplementary file 1 [file DataSheet1.zip › Original data-WB/bcl2-Original data.JPG]

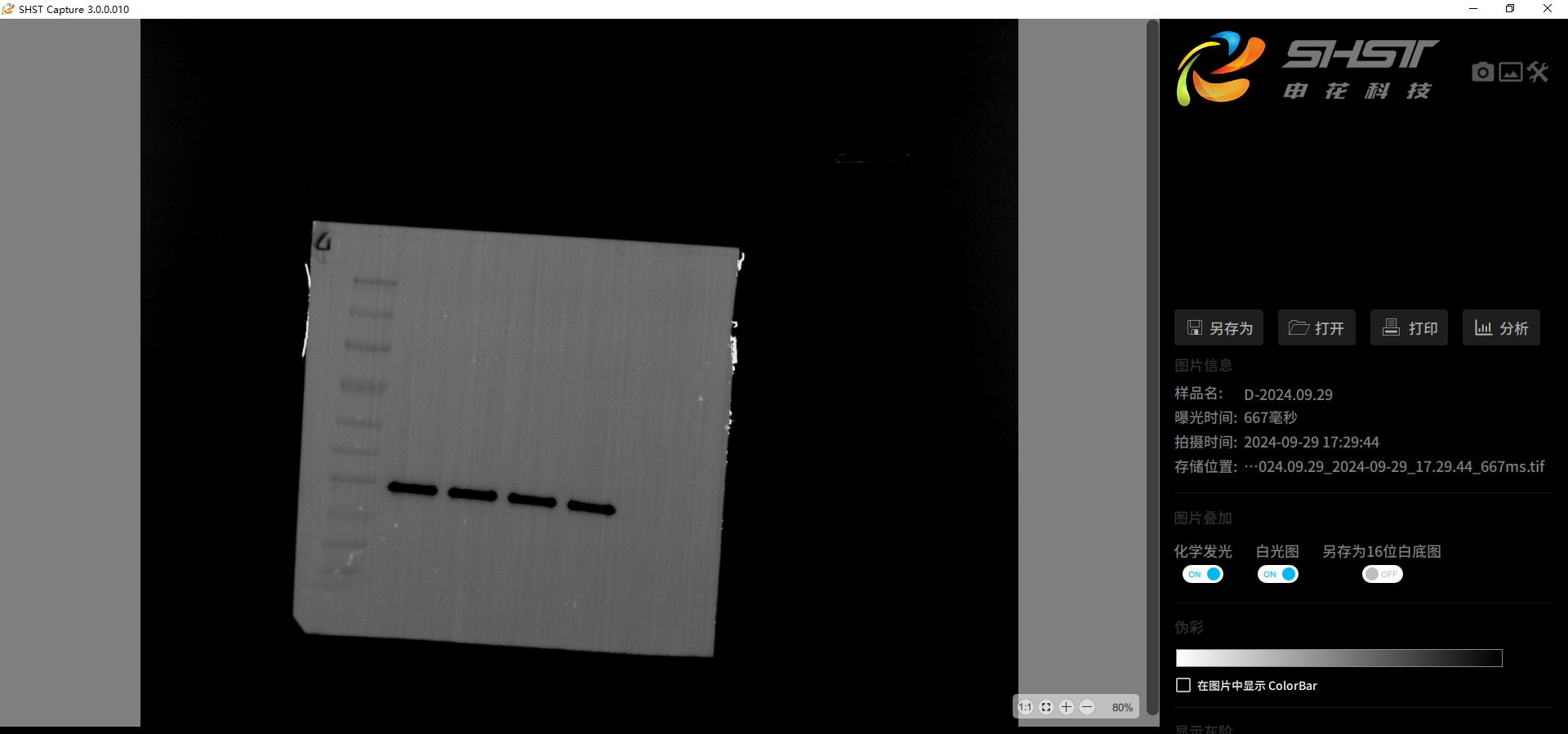

Supplement: Supplementary file 1 [file DataSheet1.zip › Original data-WB/GAPDH-Original data.JPG]

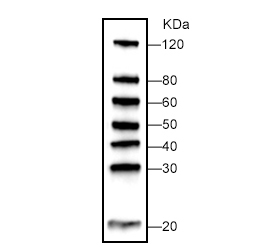

Supplement: Supplementary file 1 [file DataSheet1.zip › Original data-WB/M00521.tif]

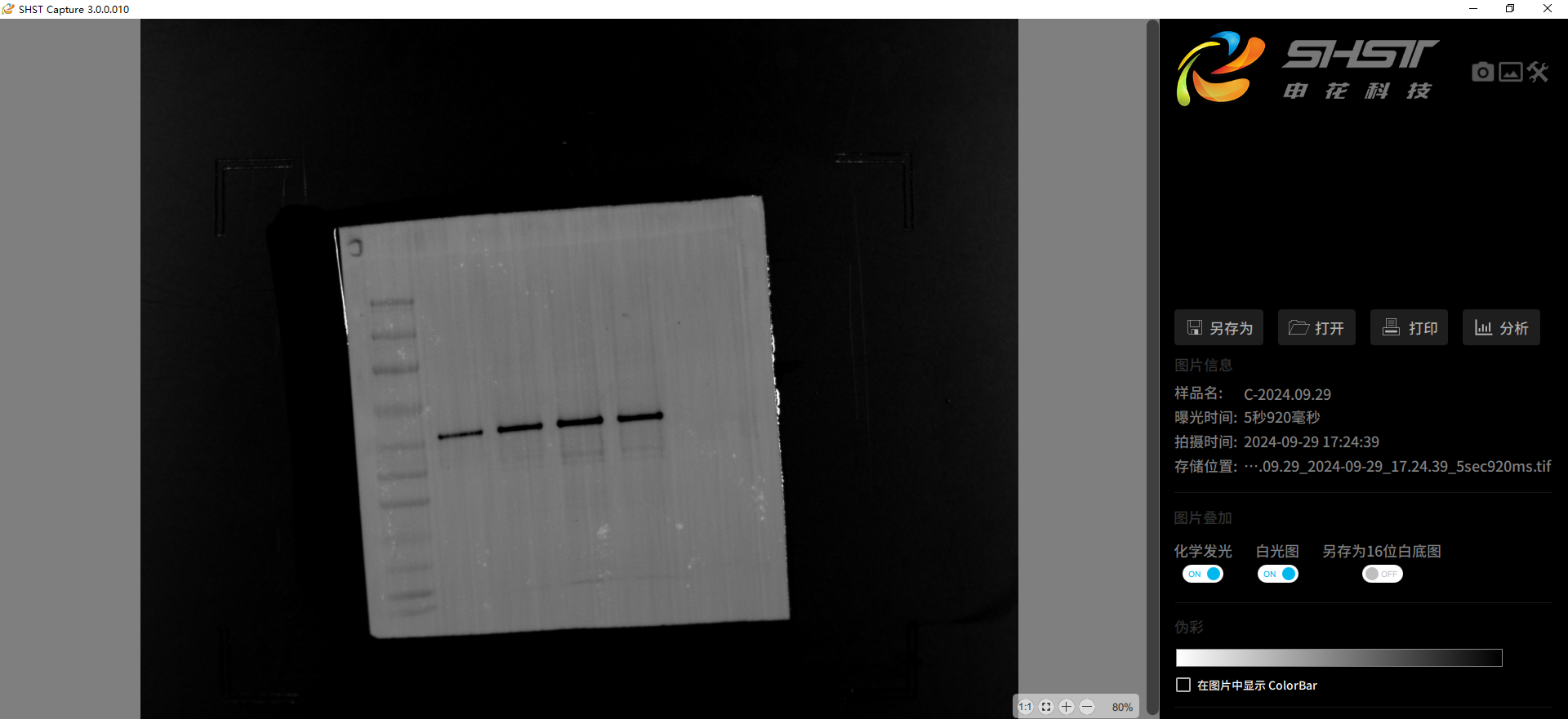

Supplement: Supplementary file 1 [file DataSheet1.zip › Original data-WB/P53-Original data.JPG]

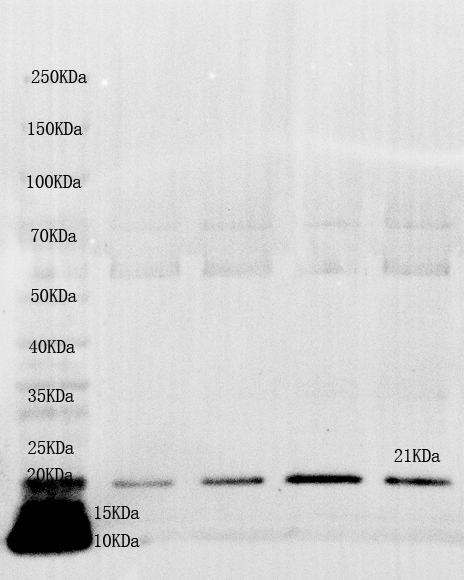

Supplement: Supplementary file 1 [file DataSheet1.zip › Original data-WB/WB-bax.jpg]

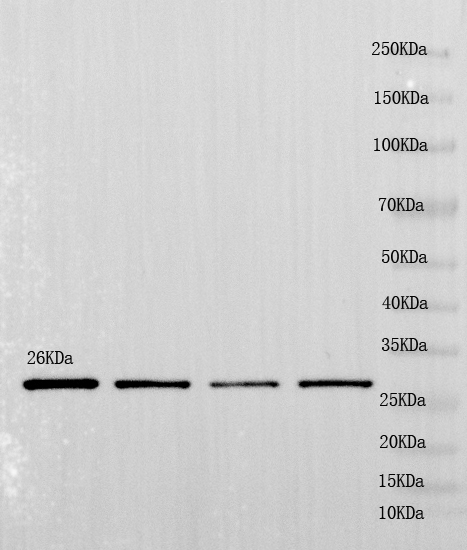

Supplement: Supplementary file 1 [file DataSheet1.zip › Original data-WB/WB-bcl2.jpg]

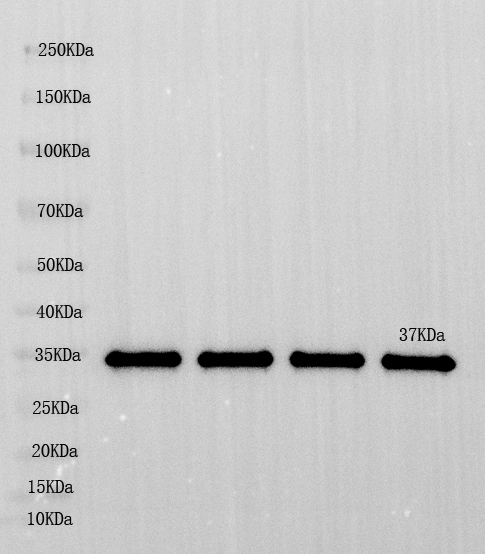

Supplement: Supplementary file 1 [file DataSheet1.zip › Original data-WB/WB-GAPDH.jpg]

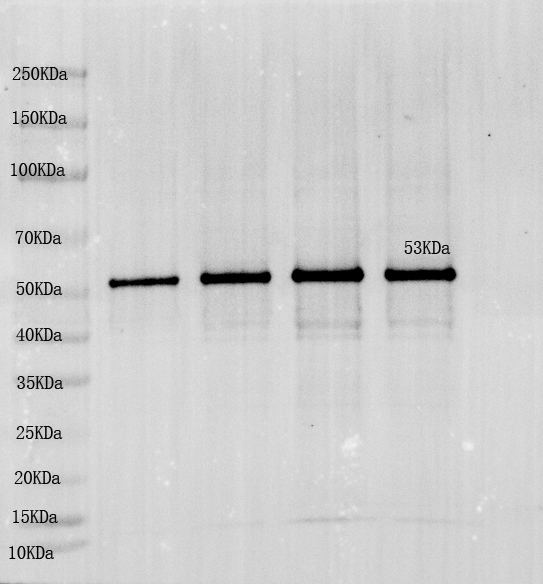

Supplement: Supplementary file 1 [file DataSheet1.zip › Original data-WB/WB-P53.jpg]
